# PW02-026 - Low frequency variants of NLRP3 in CAPS patients

**DOI:** 10.1186/1546-0096-11-S1-A167

**Published:** 2013-11-08

**Authors:** M Lesche, A Dahl, A Kränkel, J Roesler, A Rösen-Wolff

**Affiliations:** 1 Deep Sequencing Group SFB655, BIOTEC, TU Dresden, Germany; 2 Department of Pediatrics, University Clinic Carl Gustav Carus, TU Dresden, Dresden, Germany

## Introduction

Somatic mosaicism of *NLRP3* has been identified in a high percentage of “mutation-negative” patients suffering from chronic infantile neurologic, cutaneous, articular (CINCA) syndrome.

## Objectives

The aim of the study was to detect and quantify low frequency variants of *NLRP3* in German patients suffering from cryopyrin associated periodic fever syndromes (CAPS) including CINCA, Muckle-Wells syndrome (MWS), and familial cold autoinflammatory syndrome (FCAS).

## Methods

All exons of *NLRP3* were amplified by PCR (30 cycles) from genomic DNA isolated from PBMCs of healthy controls or CAPS patients. Thereafter, PCR products were concatenated, fragmented and subjected to NGS fragment library preparation followed by Illumina short read sequencing. For SNV calling a customized pipeline on basis of the GATK pipeline (1000 Genomes project) was utilized using a 40.000x coverage to assure sufficient sensitivity. In order to determine the accuracy of quantification, PCR products containing a known heterozygous mutation (T348M) were mixed with *NLRP3* wildtype PCR products to obtain dilutions of the mutated sequences of 25%, 12.5%, and 6.25%.

## Results

We were able to exactly quantify the diluted low frequency mutation (T348M). In one CINCA patient a new variant (L359S) was detected in 30% of the DNA sequences that had not been identified by classical Sanger sequencing of an older sample. Up to now we could not detect low frequency *NLRP3* variants in MWS or in FCAS patients.

## Conclusion

Massive parallel sequencing is a reliable method to quantify low frequency variants of *NLRP3*. A new *NLRP3* mutation could be detected in a patient suffering from typical CINCA syndrome. Somatic mosaicism may be less frequent in MWS and FCAS patients. Due to the fact that more “mutation-negative” CAPS patients need to be characterized, we will continue with this study.

## Disclosure of interest

M. Lesche: None Declared, A. Dahl: None Declared, A. Kränkel: None Declared, J. Roesler: None Declared, A. Rösen-Wolff Grant / Research Support from: Novartis Pharma GmbH, Nürnberg, Germany

